# Giving voice to food insecurity in a remote indigenous community in subarctic Ontario, Canada: traditional ways, ways to cope, ways forward

**DOI:** 10.1186/1471-2458-13-427

**Published:** 2013-05-02

**Authors:** Kelly Skinner, Rhona M Hanning, Ellen Desjardins, Leonard JS Tsuji

**Affiliations:** 1 School of Public Health and Health Systems, University of Waterloo, 200 University Avenue West, Waterloo, ON N2L 3G1, Canada; 2 Department of Geography and Environmental Management, University of Waterloo, 200 University Avenue West, Waterloo, ON N2L 3G1, Canada; 3 Department of Environment and Resource Studies, University of Waterloo, 200 University Avenue West, Waterloo, ON N2L 3G1, Canada

**Keywords:** Canada, First Nations, Food security, Nutrition policy, Poverty, Remote, Coping strategies

## Abstract

**Background:**

Food insecurity is a serious public health issue for Aboriginal people (First Nations [FN], Métis, and Inuit) living in Canada. Food security challenges faced by FN people are unique, especially for those living in remote and isolated communities. Conceptualizations of food insecurity by FN people are poorly understood. The purpose of this study was to explore the perceptions of food insecurity by FN adults living in a remote, on-reserve community in northern Ontario known to have a high prevalence of moderate to severe food insecurity.

**Methods:**

A trained community research assistant conducted semi-directed interviews, and one adult from each household in the community was invited to participate. Questions addressed traditional food, coping strategies, and suggestions to improve community food security and were informed by the literature and a community advisory committee. Thematic data analyses were carried out and followed an inductive, data-driven approach.

**Results:**

Fifty-one individuals participated, representing 67% of eligible households. The thematic analysis revealed that food sharing, especially with family, was regarded as one of the most significant ways to adapt to food shortages. The majority of participants reported consuming traditional food (wild meats) and suggested that hunting, preserving and storing traditional food has remained very important. However, numerous barriers to traditional food acquisition were mentioned. Other coping strategies included dietary change, rationing and changing food purchasing patterns. In order to improve access to healthy foods, improving income and food affordability, building community capacity and engagement, and community-level initiatives were suggested.

**Conclusions:**

Findings point to the continued importance of traditional food acquisition and food sharing, as well as community solutions for food systems change. These data highlight that traditional and store-bought food are both part of the strategies and solutions participants suggested for coping with food insecurity. Public health policies to improve food security for FN populations are urgently needed.

## Background

Food insecurity is a serious public health issue for Canada’s indigenous population [1,2]. The term “Aboriginal people” describes the three groups that comprise Canada’s indigenous population; First Nations [FN], Métis, and Inuit. First Nations are the largest of these three groups, making up nearly 60% of the Aboriginal population living in Canada with approximately 60% of FN people living off-reserve lands. Data on food security in off-reserve Aboriginal people are alarming; 24% of Aboriginal households had a compromised diet (reduced quality and/or quantity), and 33% experienced food insecurity compared to 8.4% and 9%, respectively across the rest of Canada [3-5]. In on-reserve FN households and Inuit households in Arctic communities the prevalence of food insecurity appears to be even higher. The First Nations Regional Health Survey found just over half (54.2%) of households surveyed were food insecure, while the Inuit Health Survey conducted in 36 Arctic communities found a range of household food insecurity from 45-69% depending on region [6,7]. Food insecurity in Aboriginal households in Canada has been associated with high levels of poverty, multi-child households, low levels of education attainment and labour force participation, reliance on social assistance/welfare, and female lone-parent households [5].

Food security challenges faced by Aboriginal people are unique [1,8-12], especially for Aboriginal people living in remote and isolated communities. Aboriginal food systems are primarily characterized by two avenues of food provision: the harvesting, sharing and consumption of traditional (or country) foods and the purchasing and consumption of market (or commercial or store-bought) foods [11,12]. Food harvested from the wild by FN people is called “traditional food” while the Inuit call wild-harvested food “country food”. Despite the combination of the traditional food system and market food system as being distinct from the non-Aboriginal food system, current conceptualizations of food security lack the context, food practices, and perspectives of Aboriginal people [1].

Regardless of evidence that food insecurity is prevalent in Aboriginal communities, little information is known about the characteristics of the individuals or households experiencing this problem [13]. While numerous food system studies have been published on Inuit people living in the Canadian Arctic in recent years [7,14-23], there are still few food system studies with on-reserve FN communities [12]. Many gaps remain about the nature and extent of food insecurity for FN people in Canada. Lead authors in this field [1,2,5] have recommended qualitative studies to better understand the food security situation for FN people. The knowledge gained can help to tailor food security programs and policies to the unique needs of these communities and population [1,5].

In this study, we explored food insecurity from the perspective of FN adults living in a remote, on-reserve subarctic community in northern Ontario, Canada. Previous work by our group had identified a high prevalence of household food insecurity (70%) using the Household Food Security Survey Module [4,24]. The intention of this study was to determine participants’ perceptions of food security and the range of adaptive strategies they use at an individual and household level. The two research questions addressed by this study were: (1) “What are the coping strategies for food insecurity used by community members?”; and, (2) “What suggestions do they have to improve food security in their community?”

### Community profile and study population

This study was conducted in Fort Albany First Nation which is situated on the west coast of James Bay in the Mushkegowuk Territory along the Albany River in northern Ontario, Canada. As described previously [25,26], the Fort Albany reserve is home to approximately 850 people. Fort Albany is geographically remote (52° 15′ N; 81° 35′ W); it is accessible only by plane year-round, by boat and barge during the ice-free season, and by ice road after freeze-up. In Canada, a remote community is defined as being more than 350 kilometres from the nearest service centre (or city) having year-round road access. Fort Albany also is categorized as a community with “special access” which means that it is located in a zone where there is no year-round road access to a service centre. Timmins, Ontario is 769 kilometres from Fort Albany and is one of the closest cities with road access. Timmins is considered a main entry point for food distribution to Fort Albany as food is flown from there during most of the year with the exception of 6–8 weeks in the winter when the ice road allows for accessibility to closer communities. One of the communities accessible by ice road is Moosonee, which is 128 kilometres southeast of Fort Albany and has train access. As a result of being remote with special access, transportation of goods into the community of Fort Albany, including commercial food, is very expensive.

At the time of this study, the community had one main grocery store and two small convenience stores. Although traditional foods remain an important part of their diet, the majority of dietary intake is from store bought food. Community members participate in traditional harvesting activities (also referred to as traditional food acquisition) including hunting, fishing, and gathering food from the land. However, these activities have been declining in recent decades, especially for young people. As these endeavours are seasonal, are limited by financial constraints for harvesting transportation and equipment, and the yield varies greatly depending on the success of the harvest, there is much variability in the consumption of traditional foods between households and over the course of the year. Traditional foods commonly harvested and consumed include berries (e.g., ground berries - *Gaultheria procumbens*), fish (e.g., whitefish - *Coregonus clupeaformis*), large land-based animals (e.g., moose - *Alces alces*), game birds (e.g., goose - *Branta canadensis interior* and *Anser caerulescens caerulescens*), and small game (e.g., hare - *Lepus americanus*). Community members live in small houses and many households have extended family living together.

Fort Albany First Nation was an ideal location for this project for a number of reasons: we have established a community advisory committee with broad community representation; have good rapport with the community and school as we have been working on school programs for healthy eating and physical activity for many years; and community members have a keen interest in improving the dietary habits of their population [25-28].

## Methods

This study builds upon our previous work where community focus groups and individual interviews with Fort Albany community members identified food insecurity as a constraint to healthy eating in children and youth [25,27]. This study was also part of a larger project to examine food security in the community. The theoretical framework for the larger project was based on systems thinking and a critical social theory perspective [29]. Critical social theory includes aspects of theories from feminism, postcolonialism, and Indigenist critical theory which better reflects Indigenous ways of knowing than a purely postcolonial approach [29]. This theory allows for a more holistic spiritual viewpoint and represents an Indigenous research paradigm that supports self-determination. The theory also focuses on specific language used by participants as a source of information about meaning [29].

### Community advisory committee

A community advisory committee (CAC) of six community members representing local stakeholder organizations (e.g., Band Council, Health Centre, school) and parents and the community at large was established prior to the study. For this study, members of the CAC were involved in the design of data collection tools (e.g., qualitative questions and probes), helped to adapt approaches to decrease the cultural sensitivity of study methodologies, assisted in collecting data, provided input on the interpretation of results, and assisted with the dissemination of results. Although a trusting relationship between the investigators and members/organizations in the community has been previously established, the specific formation of a CAC was fundamental to this participatory research. Ethics approval for this study was obtained from the Office of Research Ethics at the University of Waterloo and permission to conduct this study was obtained from Fort Albany First Nation.

### Question development

As part of the larger project, the first part of each interview began with having the participants respond to the 18-item US Household Food Security Survey Module (HFSSM). The HFSSM is a government questionnaire that was used by Health Canada in the 2004 Canadian Community Health Survey (Cycle 2.2) to determine the prevalence and severity of food insecurity in the off-reserve population [4]. The qualitative interviews for the current study were conducted following completion of the HFSSM, which is why the first interview question refers to the government questionnaire.

Lambden and colleagues [30] concluded that traditional food attributes must be included in studies of food security in the Arctic. Although this study was in the subarctic, it is apparent from previous work with Cree in the western James Bay region that questions related to traditional food are very important for studying food security in this community. These considerations were taken into account during the question development for the qualitative interviews.

A set of three questions and probes were developed using an iterative process: 1) Initially, nine questions were informed by the qualitative food security literature, including studies with Aboriginal populations in Canada (e.g., [15,22,31]); 2) During subsequent drafts, questions were reviewed to ensure they were more understandable, culturally appropriate and relevant to the food security issues in FN communities based on consultation with and input from members of the CAC (n = 3) and the investigators’ (n = 3) personal experiences with the people and community of Fort Albany. The number of questions for the final draft (n = 3) was kept small to reduce the response burden for the participants. The final three questions were: (1) The government questionnaire we did seemed to ask mostly about store-bought foods. Can you tell me about traditional foods and your household?, (2) How do you adapt if there doesn’t seem to be enough food (traditional or store-bought) for your household?, and (3) What do you think can be done to make it easier for people in Fort Albany to get enough (healthy) food (store-bought and/or traditional food)?. Table 1 displays the final three questions and probes.

**Table 1:** Interview questions and probes

**Questions**	**Probes**
Q1. The government questionnaire we did seemed to ask mostly about store-bought foods. Can you tell me about traditional foods and your household?	Probe for
	• Any barriers to accessing traditional food
	• Has environmental change affected access to traditional foods
	• Any methods to increase traditional food access and consumption
Q2. How do you adapt if there doesn’t seem to be enough food (traditional or store-bought) for your household?	Probe for
	• e.g., things you might do to make food last longer, other sources of food
Q3. What do you think can be done to make it easier for people in Fort Albany to get enough (healthy) food (store-bought and/or traditional food)?	Probe for
	• Community (community), band (government), band council (government), school (community, individual), people (individual)
	• Food sharing between community members and families

### Participant recruitment, consent, and data collection

A local community research assistant was hired to collect the data. He had been employed by our research team in the past and trained in proper protocols for data collection, including the administration of surveys and conducting interviews. He had an understanding of the project’s aims, was instructed to provide probes when appropriate, and was familiar with the practice of active listening during interviews [32]. The community assistant spoke Cree, which was helpful if any of the participants requested Cree translation. The assistant was also a member of Fort Albany First Nation, had stature in the community, and has lived there for more than 25 years. Therefore, the assistant had full understanding of the language and culture of the respondents as well as established trust and rapport; all of which are important elements for conducting qualitative interviews [33]. The status of the interviewer was very important for the comfort of the participants in discussing the sensitive topics around food insecurity.

One adult in each of the on-reserve FN homes in Fort Albany was approached in-person by the community research assistant to participate in the study. Participants were provided with an information/recruitment letter and/or the study was explained orally. Participants were given the option to be interviewed in their language of choice and their location of choice. As in our previous studies, verbal consent was obtained from all participants, being culturally appropriate for the Western James Bay region for this type of project [27,34]. Semi-directed, in-depth interviews were conducted with each willing participant from June 2009 to January 2011. Interviews were guided by the three open-ended questions and the probes were only used to prompt more discussion if the participant needed examples to stimulate the conversation. The CAC decided that due to the sensitive nature of the topic of food insecurity it was not appropriate to audio-record the interviews. The community research assistant took verbatim handwritten notes during interviews. Demographic characteristics of the respondent and household were also collected. Interviews were coded by number for anonymity and to maintain confidentiality of respondents. All willing respondents participated in the interview regardless of whether their household was classified as food secure or food insecure.

The university research team was in regular contact with the community assistant either in-person or by telephone to discuss progress on data collection and to answer any questions that might come up related to the data collection. Interview transcripts were periodically returned to the research team in batches as they were completed.

### Data management and analysis

The handwritten interviews were transcribed verbatim. Organization and coding of the transcribed data for the qualitative analysis was conducted both by hand and using QSR NVivo® computer software (NVivo, version 8.0; Doncaster, Australia: Sage Publications Software, 2008). Thematic data analyses were carried out according to the stages and steps described by Boyatzis [35] and followed an inductive, data-driven approach [35]. Initially, the raw data were reduced into logical and meaningful segments on paper [36]. Subsequently, data were organized into groups, and “themes” [37] were identified within a subsample of data from ten randomly selected interviews. The themes were then compared across subsamples and codes were created. Creating the codes was an iterative process of the writing, editing, and reconstruction of statements from the preliminary themes into a set of revised themes using the qualitative techniques of the constant comparison method and searching for deviant cases [38]. The qualitative analysis continued until saturation was reached, where no new themes emerged from the data. This also was an indication that there were a sufficient number of interviews and the sample size was adequate [39]. During the process of analyses, the theme and subtheme labels were created based on an interpretation of the statements and phrases of the participants. Therefore, the thematic labels presented in the study findings are not a verbatim representation of the exact words spoken by the participants during the interviews. Memos were used to record thoughts and ideas about the codes during the process of code development [40]. The codes were then used to analyse the rest of the qualitative data from all of the interviews. Initially the three interview questions were coded separately. Then the analysis was opened and coded across the three questions as the analyst found that the questions asked “triangulated”. The final codes were arranged into a hierarchical coding list.

Initial thematic analysis was conducted by the lead author. To determine the consistency of judgment of the coders and to establish inter-rater reliability [41,42], the codes for the themes and subthemes were confirmed by a second independent analyst who had documented expertise with qualitative methods and analysis and expertise on the topic of food security [43,44]. The second analyst applied the themes to a subset of the data which was a random selection of 50% (n = 25) of the interviews. The percent agreement between the two coders was 83%. The second coder also recommended the reorganization and addition of two subthemes and one main theme. The additions of these themes were discussed between the two coders and it was agreed that they should be included in the analysis. The final code included 10 themes and 39 subthemes.

Final emergent themes and subthemes were shared with a few of the interviewed participants (n = 5) by the lead author to confirm that they accurately reflected their perspectives. A listing of the themes and subthemes was discussed with members of the CAC and revisions to the wording of the final themes and subthemes were suggested. Sharing the themes with participants and the CAC was a form of member checking to verify results of the study [45].

## Results

Of the 76 individuals approached to participate in this study, 10 declined participation in the larger study, and 15 refused to participate in the interviews, resulting in a response rate of 67%. A total of 51 respondents participated in the interviews, 27 male and 24 female with an average age of 43.7 years. The main reason 15 individuals chose not to participate in the interviews was respondent fatigue as they had already completed the HFSSM questionnaire. Just over half of the non-participants were male (n = 8, 53%) and lived in households categorized as food secure (n = 8, 53%) and their average age was 41.3 years. Personal and household characteristics of the participating study population are shown in Table 2. Although more than three-quarters of the 51 participants had a salary from employment as their main source of income, 75.5% lived in households that were classified by the HFSSM as food insecure.

**Table 2:** Personal and household characteristics of participants (n = 51)

	**N**	**%**
Personal characteristics of participants		
Age in years		
21-30	3	5.9
31-40	16	31.4
41-50	22	43.1
51-60	10	19.6
Sex		
Male	27	52.9
Female	24	47.1
Highest level of education		
Elementary graduate or less	17	33.3
Secondary graduate or some secondary	19	37.3
Post-secondary graduate or some post-secondary	15	29.4
Main source of income		
Salary/wages from employment	39	76.5
Social assistance or other^a^	12	23.5
Household characteristics of participants		
Household food insecurity^b^		
Food secure household	12	24.5
Moderately food insecure household	27	55.0
Severely food insecure household	10	20.5
Household type		
Couple with children	31	60.8
Couple, no children^c^	11	21.6
Lone parent^d^	7	13.7
Other^e^	2	3.9
Children < 18 years living in household		
None	13	25.5
1 or 2	25	49.0
3+	13	25.5
Number of families^f^ living in household		
1	27	52.9
2	24	47.1
Total number of people living in household		
1-3	15	29.4
4-6	33	64.7
7+	3	5.9

^a^Respondents could choose other sources of income, including worker’s compensation/employment insurance, pension/senior’s benefits or any other source (e.g., alimony, child tax benefits, etc.), however all respondents without a main income source from salary/wages chose social assistance.

^b^Based on participant responses to the Household Food Security Survey Module (HFSSM). The n = 49 as two individuals chose not to respond to the HFSSM questionnaire.

^c^Includes couples living alone or those with children > 18 years.

^d^Includes lone parents living with at least one child < 18 years.

^e^Includes unattached individuals not living with any children < 18 years.

^f^A “family” was defined according to the Statistics Canada definition for Census Family [46] (Statistics Canada, 2012).

**Figure 1:**
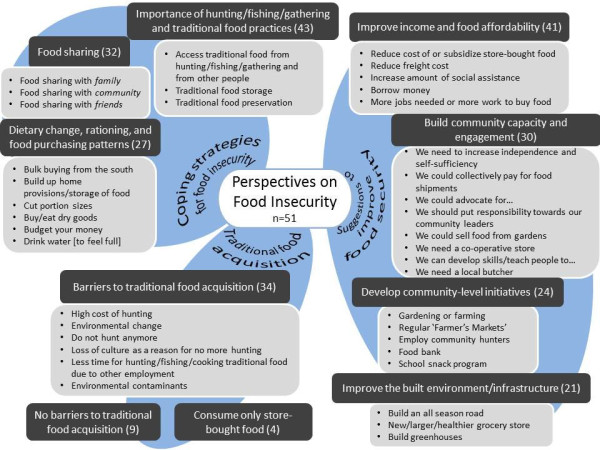
**Themes and subthemes that emerged from the thematic analysis of participant perspectives on food insecurity.** The themes are the darkly shaded boxes where the number in parentheses denotes the number of times each theme was represented by a quote. The subthemes under each theme are listed in order according to the number of participants with at least one quote for the subtheme.

The results section documents an interpretation of the findings based on the three interview questions that were asked and selected discussions that followed between participants and interviewer. Figure 1 illustrates the hierarchical coding list of themes and subthemes that represent the participant’s perspectives on food insecurity. Although there was some overlap between the questions asked, the interviews generally fell into the three question categories: traditional food acquisition; coping strategies for food insecurity; and suggestions to improve food security. For the remaining Results section, the paragraph headings are the themes. The full list of themes and subthemes are depicted in Figure 1.

### “Can you tell me about traditional foods and your household”

#### Barriers to traditional food acquisition

When describing traditional food acquisition for their household, the majority of participants reported various barriers that prevented them from acquiring traditional food on a regular basis despite their desire to eat game meats more often. The two main barriers reported were the high cost of hunting and environmental change affecting their ability to hunt in specific areas and during specific times. The high costs were attributed primarily to the cost of fuel to travel to hunting sites and the financial means to own hunting equipment. Hunting equipment included vehicles for transportation, guns, and ammunition. A number of participants pointed out that even if money was spent to go out hunting, there was no guarantee that the hunt would be successful.

*“It costs money to go hunting – gas, snow machine, and you need that money for everything else because everything is expensive.”* (Interview 4, female)

*“Gas is expensive…$1.85 a litre. But you can spend all the money to go out hunting and come back with nothing. So you’ve spent your money, but still have no food.”* (Interview 25, male)

*“Still go hunting, geese, moose, caribou but the seasons are changing and it gets harder to get wild food. For spring hunting – geese fly at odd times now. In April it is still too cold, snow and sometimes it gets too warm and snow melts too fast. Geese won’t land where there is no snow. I guess we have to change our hunting season [May and June] and go hunting at the Bay instead of the muskeg [swamp]. We try to get 12 geese at least and cook one per month.”* (Interview 17, male)

Other categories of barriers mentioned included the loss of culture as a reason for no more hunting; less [personal] time for hunting, fishing and cooking traditional food due to employment, and concern about environmental contaminants in hunted food.

#### No barriers to traditional food acquisition

A few participants did express that they thought there were no barriers to traditional food acquisition. However most of these people also admitted that they did not hunt, did not prefer game meat, or consumed only store-bought food.

### “How do you adapt if there doesn’t seem to be enough food for your household”

#### Importance of hunting/fishing/gathering and traditional food practices

This importance of traditional food acquisition and traditional food practices was a prevalent discussion topic throughout the interviews and was the predominant theme across all three of the interview questions. In addition to describing their access of traditional food from hunting, fishing, and gathering for themselves, the participants also mentioned accessing food from other people and the importance of food practices such as storing and preserving traditional food for future consumption.

*“As for me, I don’t really eat traditional food only when family member gives me wild meat. Would be nice to eat traditional food every supper.”* (Interview 46, female)

#### Food sharing

When asked how they adapt when there wasn't enough food, the majority of participants mentioned food sharing. Food sharing with family was the most common, followed by food shared between community members and then food shared with friends. Food sharing with family included immediate family as well as relatives, even if the relatives lived in another community. Food sharing was seen as a normal part of daily life and occurred more often during hunting seasons when game meat was made available by hunters. Most of the participants described that the food shared was traditional game meats.

*“We have get-togethers as family and we go to another family house and we share what we have in our homes and the other family does as well.”* (Interview 29, male)

*“Our parents have a lot of wild meat and share with our family.”* (Interview 19, female)

*“One way is to give food if other families can’t afford to buy what they run out of*.” (Interview 45, female)

#### Dietary change, rationing, and food purchasing patterns

Participants explained how they would change their eating and food purchasing patterns in times where they didn’t have enough food or when they couldn’t afford to purchase certain foods. They described how they bought food in bulk from the south and stocked up their provisions of non-perishable food when the winter road was usable. Some mentioned reducing portion sizes and changing their consumption patterns. For example, buying dry goods like rice and pasta which were cheaper to purchase.

*“We buy more in Moosonee during the winter months. Buy foods such as macaroni, rice, other dry food.*” (Interview 2, male)

### “What do you think can be done to make it easier for people in Fort Albany to get enough (healthy) food”

#### Improve income and food affordability

When participants were asked to suggest ways to make it easier for community members to get enough food, the majority pointed to the high prices of food at the local store and the low incomes of community residents. They thought that the store food should be reduced in price to become more affordable and that freight costs for shipping food should be decreased. Increasing income by increasing the amount of social assistance (welfare) payments or having more employment opportunities were also mentioned.

*“Lower cost of food would be nice so all people can afford, especially welfare recipients.*” (Interview 5, male)

#### Building community capacity and engagement

Many participants discussed the importance of increasing independence and self-sufficiency so that they didn’t have to rely on food transported from the south. They wanted to advocate for food security initiatives and put some of the responsibility for improving food security towards community leaders. For example, the Band and Band council were frequently mentioned and were viewed to be in a position to make positive changes towards increasing food availability and affordability. Many participants said “the Band should do this…”. The participants used terms that indicated a collective movement whereby community members could work together towards solutions. For example, many respondents used the words “we could…” or “we should…” or “we need…”.

*“As for the last part of the question, one method is to move back in the upper bush land (upriver) and not depend on the white society. But…we, the First Nations in the communities have already accepted everything from the start…like the signing of the treaty or letting our kids go in the residential school to get educated…we need to be independent and start doing things for ourselves.”* (Interview 52, female)

*“[There should be] scheduled hunting trips where gas and supplies are paid [by the Band] and traditional food/meat caught given to lower income families – salary for the hunter. This would provide jobs and feed the lower income groups. Lots of great hunters and trappers in this community – utilize them*.” (Interview 49, female)

#### Community-level initiatives

Participants also pointed out a number of community-level initiatives that they thought could improve food security. The most common community initiative suggested was related to gardening or farming. Community members were reminiscent about times in the past when they gardened or remembered when there used to be farming activity in the community.

“*Start a garden. You could grow things like rhubarb for jam or pies. We used to have a garden and we grew potatoes. We used the potatoes at the goose camp. We know that you can grow things here*.” (Interview 50, female)

Community members also were keen to continue and support the ‘Farmer’s Markets’ that had been started by a few community food champions. The ‘Farmer’s Market’ was an event initially held once every few months where food was purchased from a southern store and a plane was chartered to fly the food into the community. The food was then sold to community members at prices that covered the cost of the food and freight with no profit. These events were organized by a few people in the community who wanted to improve food access and affordability for community residents.

“*Okay, as for me I think they should hold more Farmer’s Markets*.” (Interview 47, female)

Another suggestion was to employ community hunters. This was seen as a way to increase the harvesting of traditional foods as well as income support for community members who were willing and keen to hunt, but might be unable to afford to hunt due to financial constraints.

*“Get Band Council to get some hunters to go hunting for spring and fall. Supply the hunters with guns, shells, gas for their trip. Whatever game [meat] is killed, it should be shared within the community.”* (Interview 51, female)

#### Improving the built environment/infrastructure

Participants made recommendations for building physical structures to promote food security and these were categorized as improving the built environment or infrastructure. They primarily mentioned the building of an all-season road in the community. An all-season road could provide year-round access to southern stores and reduce the cost of transporting food into the community.

“*Maybe an all-season road will help to have more food in cupboards, like winter time*.” (Interview 37, male)

Some participants also mentioned having a larger grocery store with more healthy food and that greenhouses should be built to grow local food in the community. The one main store in the community was regarded by residents as being too small for the size of Fort Albany, even when it was first built. In addition to housing groceries, the store also serves as the local bank and the only local business where residents can buy clothing, appliances, furniture, and electronics. Respondents expressed a desire to have better quality and more quantity of fresh fruits and vegetables as well as more fresh meat that had not been frozen. It is important to note that a small greenhouse was built at the community school over the summer and fall of 2010. The five by six meter greenhouse was partially funded through a university research grant as a case study of food security intervention strategies in the community. It was not clear from the interviews whether the respondents were motivated by the school greenhouse to suggest that more greenhouses be built or whether the idea of building greenhouses originated elsewhere.

## Discussion

Over the past century, Aboriginal populations living in northern communities have become increasingly vulnerable to the transformation of local culture and society, including a significant shift from a primarily subsistence way of life [47]. They are no longer nomadic and do not experience extreme feast or famine situations as they had in the past; however in many communities the experience of food insecurity prevails. The introduction of store-bought foods and reduction in traditional food acquisition has been a detrimental nutrition transition resulting in considerable changes to their health and well-being [11]. As northern and remote populations in Canada are continuing to be exposed to external stressors, such as environmental change; they become increasingly reliant on coping mechanisms to maintain food access [14,19]. Issues related to food security in Aboriginal populations that have not been studied in-depth include: how traditions of sharing and reciprocity of food contribute to food security; how families cope internally with food shortages; how individuals within families experience or cope with food shortages differently; how communities cope with widespread food insecurity; and what solutions or strategies have worked (or not worked) in the past and what new strategies are suggested by community members [1,2,12]. The goal of this study was to begin to explore some of these understudied issues from the perspectives of individuals living in a remote FN community. To our knowledge, this is the first study to examine coping strategies for food insecurity with a remote, subarctic FN population. The findings point to the continued importance of traditional food acquisition and food sharing as well as listening to proposed community solutions for food systems change.

### Traditional food acquisition and coping strategies for food insecurity

Similar to findings from this study, the high cost of hunting and environmental change have been cited as barriers to traditional food acquisition and affecting food security for Aboriginal people living in Canada’s north [15,19,48-53]. In this study, hunting, fishing, gathering of traditional food and traditional food practices (e.g., traditional food preservation and storage) were important ways for community members to cope with food shortages. Subsistence harvesting for Cree of the western and eastern James Bay region remains an integral part of the culture [54-59]. Outside of the clear nutritional value of wild food, the spring and fall harvesting periods constitute a cultural event which increases social and community cohesiveness. The extremely high cost of market food is prohibitive [60,61]. Thus, the contribution of wild food to the Cree diet must be preserved for both economic and cultural reasons [56]. The importance of traditional food for northern populations has been well documented [18,52,53]. Traditional food storage has become modernized with the use of freezers to store game meat for future consumption. It was not clear from the interviews whether the preservation of food, such as smoking or drying, has decreased with the increased use of freezers.

Food sharing was expressed by 63% of participants as a means of coping with food shortage. This important part of Aboriginal culture and traditions has been documented widely in the literature [6,15,19,55,62-66] including food sharing by the James Bay Cree [57,58]. The First Nations Regional Health Survey [6] found that nearly nine of ten (85.5%) respondents had traditional food shared with their household in the past year prior to the survey. Fort Albany residents felt that food sharing between family, community members, and friends was a key coping strategy when their household did not have enough food. In contrast to a few recent studies reporting that food sharing has been decreasing in northern Aboriginal communities [14,19,66], the current study found that food sharing continues to be an important way for community members to adapt to food shortages and a weakening of food sharing was not mentioned during interviews. Other studies pointing to a decline in food sharing networks cite reasons including the high cost of hunting; an increasing number of households without a hunter; and stress on hunting yields due to environmental changes [14,19,62,66]. The high costs of hunting and environmental change were both subthemes that emerged as barriers to traditional food acquisition in Fort Albany.

Dietary change, such as consuming less expensive food like rice and pasta, and rationing of food intake are coping mechanisms for food insecurity that emerged from the current interviews and are commonly cited in the literature [67-70]. Less severe forms of food rationing include cutting portion sizes to more severe behaviours such as skipping meals completely [67,71]. Participants of this study even mentioned drinking water to feel full, which indicates the severity of food insecurity for some Fort Albany residents. Specific food purchasing patterns, such as buying in bulk from more southern stores and using this practice to build up home food provisions may be unique to remote communities with seasonal road access. After freeze-up when the winter road can be safely travelled is a time of year with a great flurry of community activity as people try to buy supplies that they otherwise have difficulty accessing due to reduced availability and cost at their local store.

### Suggestions to improve food security

It must not be overlooked that the key determinant of food insecurity is poverty [72,73]. Income and food costs become more powerful determinants of food selection due to wide spread poverty and reliance on social assistance in many Aboriginal communities [2]. Suggestions to improve food security in Fort Albany reflect the reality of low incomes and high food costs in the community. However, it is positive that Fort Albany members are keen to start building community capacity and engagement and initiate community-level initiatives to improve their food security. Community-driven initiatives in FN communities tend to result in greater community buy-in and more successful outcomes.

Initiating and maintaining nonconventional agricultural initiatives have the potential for the community to increase self-sufficiency and reduce reliance on imported produce [74]. Likewise, hiring community hunters could reduce dependence on meat transported from the south and provide employment. While gardening and farming would require considerable commitment by community residents, it is already gaining momentum in the community and has benefitted from pilot-projects, including a provincially funded “Get Growing” community garden project [75] and a pilot agroforestry (local-substitution) project [74]. Community gardens have been suggested by other studies as a step towards greater food security and food sovereignty [66,74]. Harvester support programs that subsidize the cost of hunting, fishing, and trapping have been carried out in the Arctic [16,76] and subarctic [77] resulting in benefits at the community level.

In 2007 community food champions in Fort Albany began to organize a non-profit ‘farmer’s market’ event every few months. A plane would be chartered to fly in fresh and healthy food to be sold at cost to local residents. This means the prices are at least 50% lower than the same foods sold at the local grocery store because there are no overhead costs. Fort Albany has now started to call their ‘Farmer’s Markets’ an ‘alternative market’. The concept of their alternative market has begun to receive attention from a broader audience, has grown into a bi-weekly event and is being supported by external agencies that are aiming to improve northern food systems [61]. Awareness by external groups of the alternative market increased after it was presented at a national food security conference in November 2012 [78,79]. While the alternative food market does not move the community food system towards greater self-sufficiency, it can help the community take more control over food pricing of transported foods and may lead to feeling empowered for food system change.

Building an all-season road into the community was suggested by numerous participants, but is a contentious issue. On one hand, having an all-season road would mean the ability to travel by truck to other neighbouring communities - and a year-round land connection to more southerly communities - where food costs are lower even taking into account the added cost of transportation for foods imported into the community. However year-round land access could also have negative consequences, such as the greater ease of transporting drugs and alcohol. Thompson and colleagues [80] found that fly-in communities in northern Manitoba generally had more severe and higher rates of food insecurity than those with road or train access.

The participants in this study spoke about the need to increase independence and self-sufficiency with respect to accessing adequate food. They are not alone with this plea as the indigenous food sovereignty movement has been gaining momentum in Canada and has been documented through the *People’s Food Policy* project. They state that the “tribal values of giving, sharing and trading are at the heart of land care and food sovereignty” and that “the core of food sovereignty is reclaiming public decision-making power in the food system.” [81]. Fort Albany residents did not use the exact term food sovereignty during their interview dialogue, but food sovereignty was, in essence, what they were describing; they expressed a desire and suggested strategies to enhance their independence, self-sufficiency and acquisition of new skills, in addition to advocating for better food security. Food security is a precondition for, and outcome of, food sovereignty. The goal is to achieve food security concurrently with food sovereignty.

The strengths of this research include the large sample of community members that participated in the interviews and the willingness of the respondents in describing their experience of food insecurity despite the sensitivity of the topic. Participants were likely more willing to discuss their perceptions of being food insecure because of their comfort level and rapport with the local community research assistant who conducted the interviews. There were two main limitations of this study: the inability to audio-record the interviews and the generalizability of the results. First of all, although it was deemed inappropriate by the CAC to audio-record the interviews, audio-recording would have allowed the interviewer to focus all of his attention on questioning and listening during the conversation as well as capturing elements of tone and emphasis made by the participants. However, one advantage of the absence of a tape recorder is that it may have led to the relaxed nature of the interviews. Secondly, the findings in this study are not generalizable to individuals living in contexts that vary greatly in terms of food accessibility and availability. For example, those living off-reserve and in more accessible geographic locations where store bought food is considerably less expensive and where traditional food practices are not an integral and important part of the food system.

## Conclusions

Aboriginal people in Canada like the remote community of Fort Albany experience staggering rates of food insecurity and it continues to be an urgent and pervasive public health issue. Findings from this study point to the continued importance of traditional food acquisition and food sharing as well as listening to proposed community solutions for food systems change. These data highlight that traditional and store-bought food are both part of the strategies and solutions participants suggested for coping with food insecurity. The findings can be used to inform assessment and program planning activities and to advocate for policies at the local, provincial and federal levels to strengthen community food security, specifically in remote Aboriginal communities. Public health policies to improve food security for FN populations are urgently needed. While short and medium term strategies (e.g., greater employment and building community gardens and greenhouses) are important for initiating food systems change, long term sustainable food systems require policy strategies and instruments to be effective in building and strengthening food security and community capacity. Thompson and colleagues [80,82] have suggested that community economic development and supporting sustainable livelihoods through local food production and local food networks can improve food security and re-establish food sovereignty in northern FN communities.

Fort Albany was at the forefront of the Food Secure Canada conference in 2012 when the newly elected Chief of Fort Albany received a standing ovation for his emotional speech on food sovereignty in remote communities [79]. Community members in Fort Albany are speaking up about the need for food systems change, and it appears that their local leaders are listening and supporting their mission. It’s time for the provincial and federal governments in Canada to pay attention and to work with remote communities towards greater food security and to support a vision for food sovereignty.

## Abbreviations

FN: First Nations; CAC: Community Advisory Committee; HFSSM: Household Food Security Survey Module

## Competing interests

The authors declare that they have no competing interests.

## Authors’ contributions

KS, RH and LT designed and coordinated the study. KS conducted the qualitative analysis and wrote the manuscript. ED assisted with analysis. All authors read and approved the final manuscript.

## Pre-publication history

The pre-publication history for this paper can be accessed here:

http://www.biomedcentral.com/1471-2458/13/427/prepub
